# “Sleepers” and “Creepers”: A Theoretical Study of Colony Polymorphisms in the Fungus *Metarhizium* Related to Insect Pathogenicity and Plant Rhizosphere Colonization

**DOI:** 10.3390/insects9030104

**Published:** 2018-08-17

**Authors:** Steven Angelone, Iván Horacio Piña-Torres, Israel Enrique Padilla-Guerrero, Michael J. Bidochka

**Affiliations:** 1 Department of Biological Sciences, Brock University, St. Catharines, ON L2S 3A1, Canada; sa11yw@brocku.ca; 2 Division of Natural and Exact Sciences, Department of Biology, University of Guanajuato, Campus Guanajuato, Guanajuato CP 36050, Mexico; ih.pinatorres@gmail.com (I.H.P.-T.); ie.padillaguerrero@ugto.mx (I.E.P.-G.)

**Keywords:** *Metarhizium*, colony morphology, reproductive strategies

## Abstract

Different strains of *Metarhizium* exhibit a range of polymorphisms in colony phenotypes. These phenotypes range from highly conidiating colonies to colonies that produce relatively more mycelia and few conidia. These different phenotypes are exhibited in infected insects in the soil. In this paper, we provide a theoretical consideration of colony polymorphisms and suggest that these phenotypes represent a range of strategies in the soil that *Metarhizium* exhibits. We call these different strategies “sleepers” and “creepers”. The “sleeper” phenotype produces relatively greater amounts of conidia. We use the term “sleeper” to identify this phenotype since this strategy is to remain in the soil as conidia in a relatively metabolically inactive state until a host insect or plant encounter these conidia. The “creeper” phenotype is predominantly a mycelial phenotype. In this strategy, hyphae move through the soil until a host insect or plant is encountered. We theoretically model the costs and benefits of these phenotypic polymorphisms and suggest how evolution could possibly select for these different strategies.

## 1. Introduction

Phenotypic variation in fungi allows rapid adaptation to constantly changing environmental parameters and is induced by different mechanisms. The ascomycetous fungal genus *Metarhizium* is known as an insect pathogen, but it is also a rhizosphere colonizer [1,2]. This fungus also exhibits a wide variety of phenotypes when growing on agar media. A single strain can show phenotypic variation under various growth media [3] and different strains of the same species show a breadth of variation under similar growth conditions [4].

In this study, we provide examples of phenotypic variation in different strains of *Metarhizium robertsii* under a single growth regime and also show phenotypic variation of *M. robertsii* on the mummified insect cadavers. We observed a range of phenotypes growing on agar medium as well as on the insect cadaver. The extremes of the phenotypes were strains that produced many conidia while some strains produced vegetative hyphae with relatively fewer conidia. We termed these extreme phenotypes as “sleepers” (predominantly conidiating) and “creepers” (predominantly mycelial). We provide a theoretical model that the “sleeper” and “creeper” phenotypes represent different strategies for survival and host (insect or plant) seeking in the soil. We also consider the different strategies with reference to the biological “bet-hedging” hypothesis [5]. Our approach in this paper is to theoretically model the energetic costs of the “sleeper” and “creeper” phenotypes with respect to the probabilities of encountering an insect or plant host. The goal of this study is to provide a theoretical framework that would classify and explain the wide phenotypic variation observed in *M. robertsii* when infecting an insect in the soil. We also provide examples that support the theoretical model. 

## 2. Materials and Methods

### 2.1. Fungal Isolates

A total of 12 strains from Mexico and 11 strains from Canada were characterized as *M. robertsii* through DNA sequencing [6]. The strains were maintained on 25 mL of M-100 agar medium in a photoperiod of 16 h light/8 h dark at 26 °C. Conidia were harvested from cultures using sterile 0.01% Triton-X 100 and agitation with a sterile glass spatula. The resultant suspension was filtered through synthetic fiber mesh to eliminate mycelia and adjusted to a final concentration of 2 × 10^5^ conidia/mL and 5 × 10^6^ conidia/mL.

### 2.2. Sleepers and Creepers Morphology Characterization in PDA Medium

*Metarhizium robertsii* was inoculated on PDA medium (39 g/L potato dextrose broth, 1.5% agar) in duplicate with 50 microliters of a 2 × 10^5^ conidia/mL suspension in the center of each Petri dish and it was allowed to dry. One replicate was incubated in a 16:8 light/dark cycle while the other was covered with aluminum foil to simulate typical conditions in the soil. These treatments were placed in the same incubator. All samples were incubated at 26 °C for 14 days. This was done to determine that the strains used were not undergoing phenotypic degeneration or genetic defects that could affect mycelial development or conidiogenesis.

### 2.3. Sleepers and Creepers Morphology Characterization in Insects 

The proleg of each *Galleria mellonella* larvae was injected with 10 μL of a 5 × 10^6^ conidia/mL suspension. Five larvae were placed in each Petri dish over peat moss substrate (Mix 1, JVK, St. Catharines, ON, Canada), which had been previously moistened with distilled water (100 mL per 300 g of substrate) and sterilized three times. This process was repeated twice for each strain of *M. robertsii*. One plate was placed in a 16:8 light/dark cycle while the other was covered with aluminum foil and incubated at 26 °C for 14 days.

To verify that the phenotypes observed was not due to the presence of the substrate, the insect infection experiment was repeated without the use of the Peat moss substrate. In order to maintain humidity, a piece of moistened cotton was placed in the center of the Petri dish. Each Petri dish contained five *G. mellonella* larvae in duplicate. One Petri dish was used for the photoperiod treatment and one Petri dish was used for the complete darkness treatment and incubated at 26 °C for 14 days.

### 2.4. Estimation of Total Hyphal Volume

The models are designed without the use of real world data. The variables are (1) the energetic cost for hyphae to grow a certain length and (2) the probability of that hyphae to encounter a host insect or plant root. If we assume a model where individual hyphae take the shape of a cylinder with length (L), the number of cylinders would represent hyphal density (X). The energetic cost to grow a certain length will be approximated as the total volume of the cylinders.

(πr^2^L)X (1)

The probability of encountering a host would be the total volume of the hyphae divided by the volume of a sphere with an arbitrary radius of 10.

(2) $$\frac{\left(\left({\pi r}^{2}L\right)X\right)}{\frac{4}{3}\pi 10^{3}}$$

### 2.5. Effects of Passaging Creeper Phenotype

*M. robertsii* (strain BRK9), which is identified as a “creeper” was grown on PDA at 26 °C for 14 days in complete darkness. Conidia was harvested and quantified using the same technique described previously (see Section 2.1). The suspension was adjusted to a concentration of 1 × 10^4^ conidia/mL. Ten microliters of the suspension was inoculated onto minimal media agar (M-100) and 0.5× M-100 (50% concentration of M-100). These Petri dishes were covered in aluminum foil and incubated for 14 days at 26 °C. Two replicates were completed for each treatment. After the incubation period, conidia were harvested and quantified.

This process was repeated using conidia from M-100 and 0.5× M-100 agar media. This time, each suspension was passaged on M-100, 0.5× M-100, and 0.1× M-100 agar (10% concentration of M-100) and incubated under conditions previously described. A suspension of 1 × 10^7^ conidia/mL was also prepared from the initial M-100 and 0.5× M-100 agar media and 10 µL was injected into the proleg of four *G. mellonella* larvae and placed in a Petri dish containing peat moss substrate (Mix 1, JVK). These treatments were covered in aluminum foil and incubated for 14 days at 26 °C. Conidia from the second passages on artificial media were harvested and also used to inject four *G. mellonella* larvae.

## 3. Results

### 3.1. Morphological Effect in Light and Darkness

**Figure 1:**
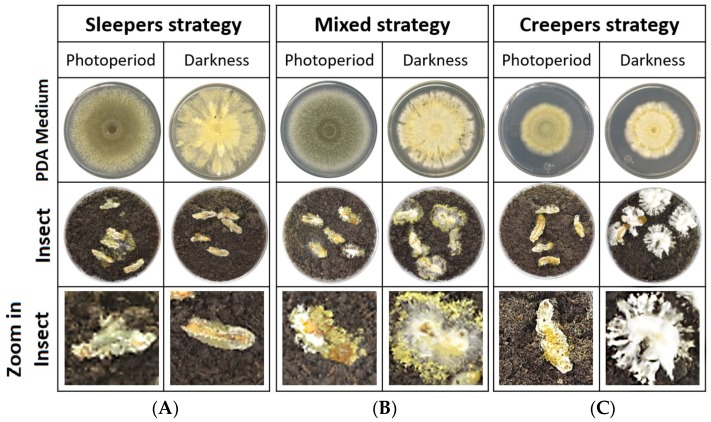
Photos of different morphologies: (**A**) sleepers, (**B**) mixed strategy, and (**C**) creepers of *Metarhizium robertsii* strains on PDA medium, infected insects, and close-up on infected insects under 16:8 light/dark and complete darkness conditions.

In complete darkness, we observed three broadly defined colony phenotypes. These are the “sleepers,” “creepers,” and “mixed strategy” phenotypes (Figure 1). The “sleeper” phenotype is predominantly a highly conidiating phenotype (Figure 1A). The “creeper” phenotype is predominantly a mycelial phenotype (Figure 1C). We also observed an intermediate phenotype and called this a “mixed strategy” (Figure 1B). “Sleepers” are visually defined as an approximate ratio of at least 3:1, conidia to mycelia, on an infected insect in the dark. A “creeper” is visually defined as an approximate ratio of at least 3:1 of mycelial to conidia on an insect in darkness. A mixed strategy is when mycelia and conidia appear in approximately equal proportions on an infected insect in complete darkness. Observations with infected insects showed that all the isolates produced conidia on mummified insects (Figure 1). Light exposure also stimulated conidiation when compared to the dark (Figure 1)*.* In addition, when grown on agar media, “creepers” exhibited noticeably smaller colony radii compared to the “sleeper” or “mixed strategy” phenotypes (Figure 1).

Neither Mexican nor Canadian strains showed any preferences for a specific phenotype (Table 1). This phenomenon was observed in Mexican and Canadian strains of *M. robertsii* and suggests a wide-ranging phenomenon that is not region-specific. 

### 3.2. Hypothetical Modeling 

**Figure 2:**
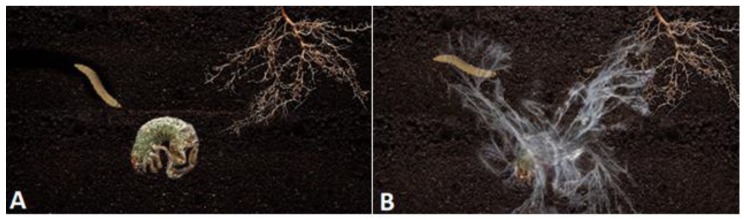
The panels show representations of insects infected by *Metarhizium* in the soil. Another insect and a plant root are shown, both of which can act as hosts for *M. robertsii*. Panel (**A**) represents a “sleeper” phenotype. The insect is predominately covered in conidia. The “sleeper” phenotype produces relatively greater amounts of conidia and this strategy is to remain in the soil as conidia in a relatively metabolically inactive state until a host insect or plant encounter these conidia. Panel (**B**) represents the “creeper” phenotype that is predominantly a mycelial phenotype. In this strategy, hyphae move through the soil until they encounter a host insect or plant.

A “sleeper” is predominately a conidiating phenotype in the dark and plays a “waiting game” strategy. An encounter with a host insect or plant would necessitate active movement by the host toward the “sleeper” (Figure 2A). Once the host is in the proximity of the fungus, the high conidial load would ensure contact with the host. In contrast, a “creeper” is primarily a mycelia phenotype and intrinsically has an increased chance of an active encounter with a host (Figure 2B). The potential advantage is a greater chance of host encounter at the expense of higher energetic cost associated with increased hyphal growth. 

**Figure 3:**
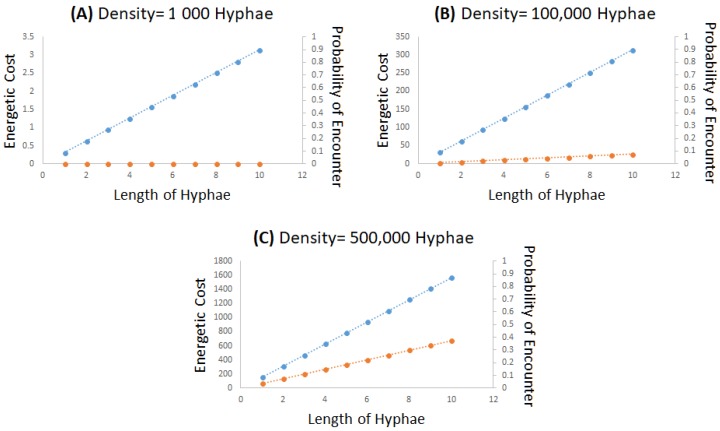
Three examples of a theoretic model relating to energetic cost of mycelial density to probability of an encounter of those mycelia with an insect or plant host. The model assumes that the cost is proportional to the total volume of the mycelia (blue line) while the probability of encounter assumes the volume of those hyphae relative to a sphere with a radius equal to the maximum length of a hypha (orange line). Shown are three examples where the mycelial density is (**A**) Low density (“sleeper”), (**B**) Medium density (mixed strategy), and (**C**) High density (“creeper”). Note that scales of the energetic cost in the three graphs are different and that there is a relatively lower metabolic cost associated with low density.

Our model attempts to represent the theoretical relationships between energetic cost and probability of host encounter as hyphal length and density increases (Figure 3). When hyphal density is low, there is a decreased probability of host encounter but with low energetic cost, which is typically representative of a “sleeper” (Figure 3A). Mycelial density and hyphal growth proportionally increase energetic cost but also increases the probability of encountering a host (Figure 3B,C). When hyphal density is greater, we suggest a greater probability of encountering a host but with a greater energetic demand, which is representative of a “creeper” (Figure 3C). The “mixed strategy” phenotype represents an intermediate density (Figure 3B). 

### 3.3. Minimal Media Effects on Creeper Phenotype

**Figure 4:**
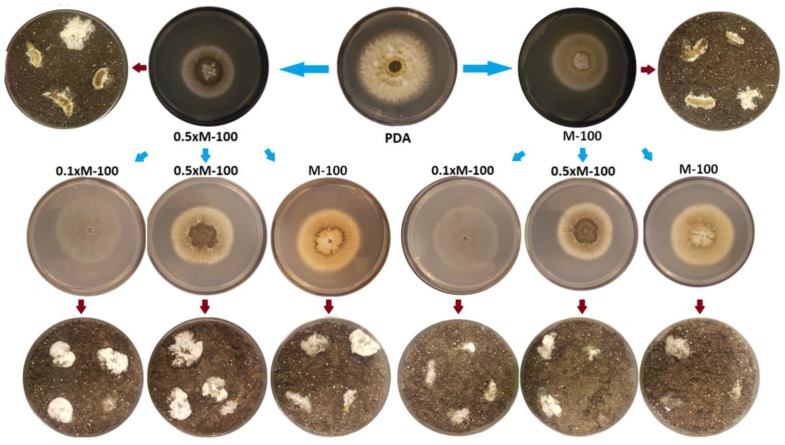
Results of passaging a “creeper” phenotype on minimal media and subsequent phenotype of the mummified insect. Red arrows represent passage from artificial media to the insect while blue arrows represent passage from artificial media to artificial media. Colony phenotype is dependent on the strength of M100. However, the “creeper” phenotype was observed in infected insects regardless of media type.

Colony morphologies of *M. robertsii* (strain BRK 9) on different media were dissimilar (Figure 4). However, passaging onto insects resulted in no differences in mummification morphologies of the infected insect (Figure 4). A second passage onto agar media showed that colony morphologies remained stable. Subsequent infection of *G. mellonella* larvae using conidia from these colonies revealed no change in mummified insect phenotype. This suggests that the “creeper” phenotype observed in mummified insects may not be influenced by previous passaging on artificial media (Figure 4).

## 4. Discussion

The ability of the rhizosphere inhabiting, endophytic, insect-pathogenic fungus, *Metarhizium*, to survive in bulk soil in the absence of a suitable host remains widely unknown. In this study, we provide a theoretical framework that suggests why there is a range of phenotypic growth patterns that potentially represent a gradient of soil survival strategies. These phenotypes were defined based upon the relative ratio of mycelia growth to conidiation in darkness and were termed “sleepers” (predominantly conidia), “creepers” (predominantly mycelia), and “mixed strategy” (approximately equal proportions of conidia and mycelia on the mummified insect). We modeled the energetic costs of the “creeper” and “sleeper” phenotypes and showed that, while the “creeper” has a greater chance of encountering a host, there is an increased energetic cost with sustained growth in the soil. Yet, the “sleeper” phenotype incurs less energetic cost but with a lowered chance of encountering a host in the soil. One hypothesis that may explain this phenomenon is biological bet-hedging [5]. Bet-hedging is a strategy where an organism sacrifices fitness in ideal conditions to have a higher fitness under stressful conditions [7,8]. These types of strategies have been identified in fungi. *Neurospora crassa* can produce ascospores with varying dormancy rates and with variable heat resistance [9]. Non-dormant ascospores germinate quickly but are easily killed by heat while the dormant ascospores are resistant to heat treatment but do not germinate easily [9]. Similarly, *Saccharomyces* can produce two different cell types, which include cells that grow quickly under ideal environments but cannot adapt very well to external stress and cells that have a slow growth rate but are more resistant to environmental stresses [7,8]. While these bet-hedging strategies reduce the fitness of the organism in any one condition, this approach allows a higher overall fitness regardless of the environment. With respect to *Metarhizium*, the “sleeper” and “creeper” phenotypes may represent bet-hedging strategies that allows survivability in the soil depending on the density of host insects and plants and/or the soil nutrient status. 

## 5. Conclusions

In conclusion, the “sleeper” and “creeper” phenotypes are defined by the proportion of conidia and mycelia produced during insect pathogenesis in the absence of light. These morphologies may be an example of bet-hedging adaptation. Uncovering the mechanisms that drive these phenotypes may reveal strategies fungi employ for survival in soil, which could have important implications regarding their persistence when utilized in biological control efforts. Currently, however, *M. robertsii* is the only species of *Metarhizium* that has been investigated and found to show this phenotype range. We are currently investigating mechanisms that control the “sleeper” and “creeper” phenotypes as well experimentally testing this phenomenon under natural conditions in the soil.

## Figures and Tables

**Table 1:** Percentage of sleepers, creepers, and mixed strategy phenotypes in complete darkness of *M. robertsii* strains from Mexico and Canada.

	Sleepers	Mixed Strategy	Creepers
Isolates Canada	3 (27.7%)	4 (36.4%)	4 (36.4%)
Isolates Mexico	5 (41.6%)	3 (25%)	4 (33.3%)
